# The combined impact of ankle–brachial index and transcutaneous oxygen pressure on mortality in patients with type 2 diabetes and foot ulcers

**DOI:** 10.1007/s00592-021-01731-9

**Published:** 2021-05-08

**Authors:** Katarina Fagher, Magnus Löndahl

**Affiliations:** 1 Clinical Sciences in Lund, Lund University, Lund, Sweden; 2 Department of Endocrinology, Skåne University Hospital, Lund, Sweden

**Keywords:** Diabetic foot, Microvascular disease, Macrovascular disease

## Abstract

**Aims:**

A diabetic foot ulcer (DFU) is associated with increased cardiovascular risk and mortality, independently of ulcer etiology (ischemic, neuro-ischemic or neuropathic). Ankle–brachial index (ABI) is the most commonly used test when diagnosing peripheral macrovascular disease and is a well-known marker for increased cardiovascular risk. Transcutaneous oxygen pressure (TcPO_2_) is considered to better evaluate microvascular function and has in previous studies shown correlations with both wound healing and survival. The aim of this study was to evaluate the combined impact of a low TcPO_2_ (<30 mmHg) and a pathological ABI (<0.9 or ≥1.4) on three-year mortality in patients with DFU.

**Methods:**

Type 2 diabetes patients aged <90 years, with at least one DFU who underwent vascular assessment with ABI and TcPO_2_ were screened for participation. The primary endpoint was mortality after three years, assessed from the National Death Registry in Sweden.

**Results:**

The study enrolled 235 participants with a median age of 76 years. Individuals with either an abnormally high or low ABI in combination with a low TcPO_2_ had the worst survival rates, with three-year mortality of 54%, compared to 42% in those with one abnormal variable (either ABI or TcPO_2_), and 21% in those with normal ABI and TcPO_2_.

**Conclusions:**

Combining ABI and TcPO_2_ when risk stratifying DFU patients seems to provide additional predictive information, not only concerning ulcer healing and limb salvage, but also on survival.

## Introduction

A diabetic foot ulcer (DFU) is a common and serious complication of diabetes, with a life-time incidence of approximate 15% [1]. It is a major cause of morbidity, reduced quality of life, and is today the leading cause of lower-limb amputations in the western world [2]. Further, a DFU has huge consequences on survival, and might serve as a marker for an advanced, general vascular disease associated with poor prognosis [3]. Individuals with DFU have a 2.5 times higher mortality risk compared individuals with diabetes but without a DFU [4, 5].

Presence of peripheral arterial disease (PVD) with a low ankle–brachial index (ABI) is a well-known risk factor for concomitant cardiovascular disease as well as increased mortality [6–8]. However, when evaluating survival among individuals with diabetes, not only macrovascular complications but also the presence of microvascular complications seem to be important predictors for survival [9]. Transcutaneous oxygen pressure (TcPO_2_) is a non-invasive method assessing tissue perfusion and is considered to better reflect the microvascular function in the skin [10].

A reduced TcPO_2_ level has in previous studies been associated with both lower ulcer healing rates and increased mortality [11, 12]. Whether a combination of a pathological ABI and a low TcPO_2_ could provide further prognostic information on survival compared to ABI alone has previously not been studied.

The primary aim of this study was to evaluate the combined impact of a low TcPO_2_ level (<30 mmHg) and a pathologically low or high ABI (<0.9 or ≥1.4) on three-year mortality in people with DFU. Secondary aims were to evaluate the combined impact of a low TcPO_2_ and a pathological ABI on ulcer healing and amputation rates.

## Methods

For this study, we screened all patients who performed a vascular assessment with Periflux 5000 diagnostic instrument (Perimed AB, Stockholm Sweden) at our DFU-unit at Skane University Hospital in Lund between years 2013 and 2015. Inclusion criteria were type 2 diabetes, age <90 years and at least one ulceration (malleoli or below). Exclusion criteria were lack of ABI or TcPO_2_ value. All patients were treated according to international guidelines regarding the diabetic foot and were referred to vascular intervention when indicated [13]. Patients were followed at our DFU-unit at a regular basis until ulcer healing or until major amputation. Ulcer healing was defined as complete epithelialization without a major amputation, and this parameter was retrospectively evaluated from patients’ medical records after 3 and 12 months. Major amputation was defined as amputation level above the ankle and data were collected from surgical records. Survival status after three years was assessed from the national death registry in Sweden.

Vascular examinations were performed with Periflux 5000 diagnostic instrument, in room temperature, with patients in a supine resting position, breathing normal air. ABI was calculated as ankle pressure divided by arm blood pressure and was measured on both legs. A threshold of <0.9 or ≥1.4 was used to define pathologically low or high ABI [13]. Before categorizing abnormally high and abnormally low ABI together as pathological ABI, we first performed separate survival analysis for these two groups. TcPO_2_ measurements were performed at the dorsum of both feet. A cut-point of TcPO_2_ <30 mmHg was used in our analyses [14]. For mortality analysis we used the lowest TcPO_2,_ and the lowest *or* highest value of ABI of the two separate legs, while the value of the ulcered leg was used when evaluating ulcer healing and amputations. Patients were stratified into three groups; group 1: both pathological ABI/low TcPO_2_, group 2: pathological ABI *or* low TcPO_2_ and group 3: normal ABI /TcPO_2_.

Baseline characteristics at the time for vascular examination, included medical history regarding prevalent cardiovascular disease (CVD) which was defined as myocardial infarction, previous coronary intervention, angina pectoris, heart failure or cerebrovascular disease. Further data on diabetes duration, smoking habits (ever), on-going medications, and laboratory data (plasma creatinine, HbA1c, lipids) were assessed from patient charts.

Hypertension was defined as blood pressure ≥140/90 or use of antihypertensive drugs. Hyperlipidaemia was defined as total cholesterol of >5 mmol/l and/or LDL-cholesterol >2.5 mmol/l or use of cholesterol lowering drugs. MDRD-equation was used to calculate estimated glomerular filtration rate (eGFR) and renal impairment was defined as an eGFR <60 ml/min/1.73 m^2^ [15].

Ethical approval was given by the regional ethical committee in Lund, Sweden, and patients were asked for informed consent.

## Statistical analyses

All statistical analyses were performed by use of SPSS version 25 (IBM, IL, USA). Baseline demographic data are presented as median and interquartile range (IQR) for continuous data, and as percentages (%) for categorical data. Comparisons between groups were performed with Kruskal–Wallis one-way ANOVA test or with Chi 2 tests. Kaplan–Meier methodology with Log-rank test was used to evaluate mortality between groups. To adjust for plausible confounders, we used Cox proportional hazard models to assess hazard ratios (HR) with 95% confidence intervals (CIs). Variables with well-known impact on mortality, such as age, previous CVD and renal dysfunction, as well as those variables that differed between groups at baseline were stepwise entered into the model. Those variables with either an independent impact on mortality or that changed the coefficient of our principal variables were then kept in the final Cox model. A two-tailed *p* value <0.05 were considered statistically significant.

## Results

Two hundred and thirty-five patients with a median age of 76.0 years were recruited in the study. Table 1 shows the baseline demographic of the study population, stratified into the three different groups of different ABI /TcPO_2_ combinations. In the group with only one pathological variable the majority (86.7%) had a pathological ABI, whereas 13.3% had a TcPO_2_ <30 mmHg. Patients with both pathological ABI and low TcPO_2_ were more likely to be female, to have renal impairment, and more likely to have an adverse cardiovascular risk profile. There were no statistical differences regarding age, diabetes duration, HbA_1c_ or hyperlipidaemia between the groups. The combination of a low TcPO_2_ level and a pathological ABI was significantly associated with worse ulcer healing rates and higher rates of major amputation during the follow-up period, as demonstrated in Table 2. The relative risk of having an unhealed ulcer after one year was 1.9 (95% CI 1.3–2.9) and the risk of having a major amputation was 4.6 (95% CI 1.8–13.2) among individuals with both pathological ABI and a low TcPO_2_ compared to those with normal values.

**Table 1:** Baseline characteristics of the study population stratified by ABI and TcPO_2_

	Normal ABI and TCPO_2_ ≥30 mmHg	ABI $<$0.9 or ≥ 1.4 or TcPO_2_ <30 mmHg	ABI $<$ 0.9 or ≥ 1.4 and TcPO_2_ < 30 mmHg	*p* value
*N* = 235	60	128	47	
Age *(years)*	75.0 (67.3–81.0)	77.0 (69.0–83.0)	75.0 (71.0–82.0)	n.s
Diabetes duration *(years)*	15.0 (6.3–22.0)	15.0 (8.0–22.3)	18.0 (10.0–25.5)	n.s
HbA1c *(% DCCT) (mmol/mol)*	7.4 (6.5–8.5) 57 (47–69)	7.6 (6.7–8.6) 60 (50–70)	7.2 (6.4–8.6) 55 (46–70)	n.s
eGFR ml/min/1.73	69 (53–92)	61 (43–86)	49 (30–64)	0.000
Sex (% females)	23.3	27.3	44.7	0.039
Insulin treated (%)	70.0	68.8	74.5	n.s
Sulphonylurea (%)	15.3	10.2	6.4	n.s
Metformin (%)	50.8	36.2	27.7	0.041
SGLT 2 inhibitors (%)	1.7	0.8	0.0	n.s
Incretins (%)	6.8	8.6	4.3	n.s
Aspirin	56.9	49.2	59.6	n.s
Anticoagulants	16.9	30.7	27.7	n.s
Smoking, ever (%)	13.5	28.8	35.9	0.035
Hypertension (%)	88.3	97.7	93.6	0.026
Hyperlipidemia (%)	87.3	85.5	90.9	n.s
CVD (%)	51.7	67.2	76.6	0.021
ABI <0.9 (%) ABI ≥1.4 (%)	0 0	73.4 13.3	91.5 8.5	0.000
TcPO2 <30 mmHg	0	13.3	100	0.000

Data are expressed as median (IQR) or percentages. *P* values <0.1 are shown; otherwise n.s is stated

**Table 2:** Differences in ulcer healing, revascularization and major amputation during follow-up in patients stratified by baseline ABI and TcPO_2_

	Normal ABI *and* TCPO_2_ ≥30 mmHg	ABI $<$0.9 or ≥ 1.4 *Or* TcPO_2_ <30 mmHg	ABI $<$0.9 or ≥1.4 *and* TcPO_2_ <30 mmHg	*p* value
Vascular intervention (%)	3.3	20.2	35.0	0.000
Endovascular (% of total)	*100.0*	*81.8*	*86.7*	
Surgical	*0*	*18.2*	*6.7*	
Both endovascular and surgical	*0*	*0*	*6.7*	
Healed within 3 months (%)	34.4	17.5	12.5	0.004
Healed within 1 year (%)	70.0	54.4	42.5	0.007
Major amputation (%)	5.5	4.4	25.0	0.000

Data are expressed as percentages

**Fig. 1:**
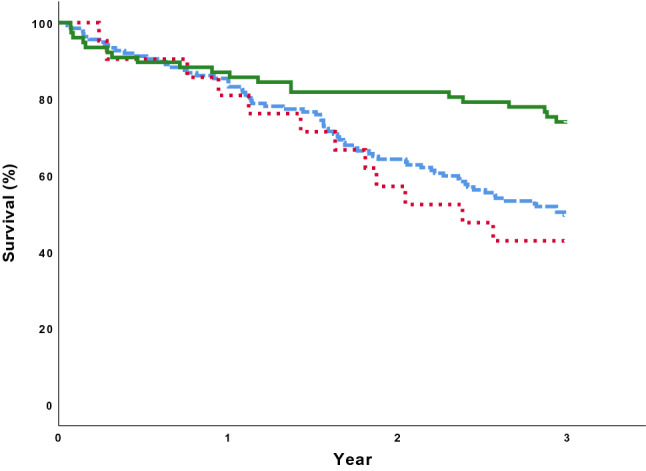
Kaplan–Meier survival curves analyzed with Log-rank test, for the primary endpoint of mortality during the three year of follow-up, within the different groups of ABI. Green solid line: Individuals with normal ABI. Blue dashed line: Individuals with ABI <0.9. Red dotted line: Individuals with ABI ≥1.4. *p* = 0.002 comparing normal ABI with either ABI <0.9 or ABI ≥1.4. *p* = 0.569 comparing ABI <0.9 with ABI ≥1.4.

**Fig. 2:**
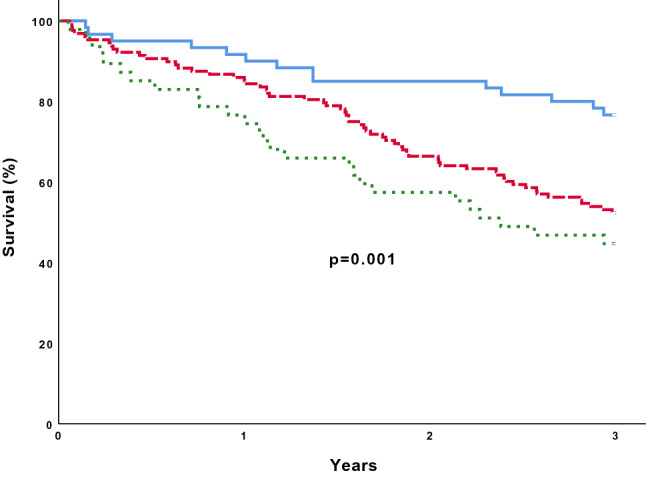
Kaplan–Meier survival curves with Log-rank test, for the primary endpoint of mortality during the three year of follow-up when combining ABI and TcPO_2_. Blue solid line: Individuals with normal ABI and TcPO_2_ ≥30 mmHg. Red dashed line: Individuals with either pathological ABI (<0.9 or ≥1.4) or low TcPO_2_ <30 mmHg. Green dotted line: Individuals with both pathologically ABI and low TcPO_2_

During the three years of follow-up, 43% of our participants died. There was no difference in mortality rate between the groups of individuals with a low ABI <0.9 compared to those with abnormally high ABI ≥1.4 (*p* = 0.569). As demonstrated in Fig. 1, both high and low ABI were significantly associated with increased three-year mortality compared to normal ABI (*p* = 0.002). The highest mortality rate was seen in the group of the individuals with a combination of either a high or low ABI together with a low TcPO_2_, as demonstrated in Fig. 2. The three-year mortality among these patients was 55.3%, compared to 47.7% in the group with only one pathological vascular parameter, and 23.3%, in those having normal ABI and TcPO_2_ levels (*p* <0.003). Cox regression models were performed to adjust for plausible confounders, such as differences in baseline characteristics and other well-known risk factors for mortality (age, gender, smoking, diabetes duration, CVD, eGFR <60 ml/min/1.73 m^2^, hypertension, revascularization, ulcer healing at three months and major amputation). Hypertension, gender, smoking, eGFR and major amputation were not independently associated with mortality, nor found to be confounders behind the results and were thus not entered in the final Cox model. The result of the final multivariate Cox model is given in Table 3. In this analysis, the combination of a pathological ABI and a low TcPO_2_ was the strongest independent predictor of mortality, with a HR of 2.19 (1.11–4.33). If only one parameter was pathological (ABI *or* TcPO_2_) no significant association was found after adjustment [HR was 1.78 (0.97–3.26)]. Ulcer healing after three months, the presence of CVD, diabetes duration and age were also independently associated with mortality.

**Table 3:** Predictors for three-year mortality based on the final multivariate Cox regression model

	Adjusted hazard ratio	95% CI	*p* value
ABI $<$0.9 or ≥1.4 *or* TcPO_2_ <30 mmHg	1.78 *	0.97–3.26	0.063
ABI $<$0.9 or ≥1.4 *and* TcPO_2_ <30 mmHg	2.19 *	1.11–4.33	0.024
Age (one year increase)	1.06	1.03–1.09	0.000
Diabetes duration (one year increase)	1.02	1.00–1.05	0.026
Ulcer healing at 3 months	0.57	0.33–0.98	0.042
Cardiovascular disease	2.11	1.21–3.68	0.008

^*^Compared to individuals with normal ABI and TcPO_2_ as the reference group

## Discussion

Prediction of wound healing and major amputation in patients with diabetic foot ulceration is clinically important both to stratify risk and target interventions for limb salvage. However, predictors for ulcer outcome might also have major impact on survival. Our study confirms that mortality among individuals with DFU is still alarmingly high, despite a high technological and improved quality health care over the last decades. Forty-three percent of our study participants died during the three-year of follow-up. This indicates a need for valuable tools for risk stratification, and a raised awareness for the diabetic foot patient. In our study, we have demonstrated that the combination of an abnormal ABI (high or low) and a low TcPO_2_, might help us to identify high-risk patients with urgent need for multifactorial intervention not only improving peripheral circulation, but also to reduce life-threatening complications. Our results indicates that the combination of a pathologically high or low ABI and a low TcPO_2_ is associated with a more than twofold increase in three-year mortality compared to normal ABI/TcPO_2_. In a German cohort of 247 patients with DFU, Morbach et al. reported a 33.1% three-year mortality in the whole DFU-population, and among those with PVD at baseline, defined by ABI <0.9 mortality was 44.1% [16]. Our mortality rate was higher, which might reflect an older study population in our study compared to Morbach´s trial (68.8 vs. 76.0 years). The high mortality rate among DFU-patients has traditionally been explained by a higher burden of traditional macrovascular complications. An ABI 0.9 correlates frequently with other coexisting macrovascular complications and has in previous studies been shown to predict both fatal and non-fatal cardiovascular events, as well as all-cause mortality [6, 8]. In our study, both high as well as low ABI seem to be risk factors for mortality, consistent with previous studies [17]. We also found that individuals with ABI <0.9 were more likely to have coexisting CVD, compared to those with normal ABI (71.5 vs. 57.1% *p* = 0.031). However, this was not seen in the group with elevated ABI (52.4%). However, the number of patients with elevated ABI was small (*n* = 21), and thus conclusions must be drawn cautiously. Further, we lack data on the separate diagnosis included in the composite CVD diagnose, and thus we cannot exclude differences in the distribution between the groups.

In DFU patients the vascular condition is often complex [9]. There are some important limitations with ABI measurements in diabetes and especially among those with neuropathy [18]. Due to medial arterial calcifications, there is a risk of falsely elevated ABIs underestimating the severity of PVD [19, 20]. Further, coexisting microvascular complications, such as diabetic kidney disease and neuropathy, often contributes to the poor prognosis both concerning ulcer healing and survival. Previous studies have shown that a low TcPO_2_ has been associated with a higher burden of other microvascular complications as well as an increased risk of major cardiovascular events [12, 21]. In our study, patients with pathological ABI and a low TcPO_2_ were more likely to have reduced renal function, result consistent with other studies [22–24]. Another often overlooked microvascular complication of diabetes that frequently co-exists in DFU-patients with distal polyneuropathy and microangiopathy is cardiovascular autonomic neuropathy (CAN). In a large meta-analysis of 15 studies including 2500 patients with diabetes, the presence of CAN was a strong predictor for mortality with a relative risk of 3.5 (95% CI 2.66–4.47) [25]. In our study, we lack quantitative data on neuropathy and CAN, which is a limitation. From other studies evaluating DFU individuals, it is likely that the prevalence of neuropathy in our population is high. For example, in the large multicenter trial EURODIALE the prevalence of neuropathy was 86% in DFU patients [26]. However, since we lack data in our trial, we can only speculate about a plausible association between TcPO_2_, peripheral neuropathy and CAN, and their role for mortality.

It is previous well demonstrated that ulcer healing is compromised in patients with low ABI as well as in those with a low TcPO_2_ [14, 27, 28]. This was also confirmed in our study. The worse healing rate and a significant increase in major amputations were seen among those with a combination of pathological ABI/low TcPO_2,_, indicating that these patients might need an even more urgent vascular evaluation. There was an unexpectedly low revascularization rate among our patients. In the group with both pathological ABI/low TcPO_2_, only 35% performed a vascular intervention. When analyzing the data, we found additional 20% who were assessed by a vascular surgeon, but intervention either failed or was considered too hazardous. Among the remaining 45% there were individuals not suitable for intervention because of multiple severe comorbidities, renal failure, and a few cases with significant ulcer improvement without vascular intervention. However, there were still a small number of patients without any rational behind why no vascular evaluation was performed. When comparing our results with others, we can conclude that our finding is not unique, indicating a general need for improvement in the management of the diabetic foot. For example, the large study EURODIALE demonstrated that 56% of the patients with critical limb ischemia performed vascular imaging, and of them, only 43% were revascularized [29]. Further, Malyar et al. reported revascularization rates of 18% and angiography rates of 25% in their study population [6]. We cannot tell whether a higher rate of vascular intervention would have had positive affect on our amputation rates and on survival. There was a non-significant trend towards better survival among individuals with pathological ABI/low TcPO_2_ who underwent a revascularization in our study. However, this could just as well be the result of a less complex comorbidity, rather than a result of the revascularization per se. In addition, neither revascularization nor major amputation rates turned out to be independent predictors for survival in our study. Similar result was demonstrated in a study by Faglia et al. In that study, 28.2% of the individuals who underwent a vascular intervention due to critical limb ischemia died, compared to 81.6% of the patients who did not undergo such procedure. However, after adjusting for several risk factors in that study, only age turned out to predict mortality and was considered a confounding factor [30]. Nevertheless, vascular intervention has profound impact on wound healing, which also turned out to be a protective factor for survival in our study.

Traditionally, male gender is considered a risk factor for developing foot ulcers and previous studies has also reported male gender as a risk factor for amputation [23]. Also, in our trial the majority (70%) of all patients were men, but one unexpected finding in our trial was the high rate of females among those with worse peripheral circulation. One plausible explanation behind this finding might be that our female participants were older (81 vs. 75 years *p* = 0.007) and had worse renal function (eGFR 54 vs. 64 ml/min/1.73, *p* = 0.017) compared with males. However, after adjusting for age and renal function, gender was not independently associated with mortality. Although our study population was relatively large, we cannot exclude a limitation in statistical power that enables us to detect small differences in other important risk factors and thus, conclusions regarding these must be drawn cautiously. Further, we cannot exclude a selection bias, as we only included individuals who were vascular examined at our department. There might thus be few patients with ulcers that healed promptly, as well as few individuals with acute limb-threatening ischemia admitted straight into acute hospital care, who might not have been vascular examined at our department, and consequently not included in our study. This could have influenced both the demographics of our study population as well as outcome. It is however unlikely, that lacking few individuals within these two subgroups would diminish the risk of a pathological ABI and TcPO_2_ on mortality. Despite these limitations, our DFU cohort represents a relatively large “real-world” cohort, with a robust primary endpoint, not accomplished with any biases.

In conclusion, our study confirms that mortality among individuals with DFU is still alarmingly high, and the need of a more aggressive approach towards cardiovascular risk factor management is crucial. Combining two non-invasive vascular screening methods, TcPO_2_ and ABI, provides important predictive information which might help us to risk stratify individuals, identifying those with urgent need for interventions not only targeting limb salvage, but also targeting survival.
